# Light-Written Reversible 3D Fluorescence and Topography Dual-Pattern with Memory and Self-Healing Abilities

**DOI:** 10.34133/2019/2389254

**Published:** 2019-11-02

**Authors:** Jing Bai, Luzhi Zhang, Honghao Hou, Zixing Shi, Jie Yin, Xuesong Jiang

**Affiliations:** School of Chemistry & Chemical Engineering, State Key Laboratory for Metal Matrix Composite Materials, Shanghai Jiao Tong University, Shanghai 200240, China

## Abstract

To achieve the dynamical dual-pattern with multiplex information of complex topography and 3D fluorescence is challenging yet promising for wide applications ranging from visual bioassays, memory, smart devices to smart display. Here, we develop a convenient, reliable, and versatile method to realize the well-ordered dual-pattern with reversible topography and 3D fluorescence via a light direct-writing approach based on the wrinkle mechanism. By introducing the charge transfer (CT) interaction between *π*-electron-rich anthracene (AN) and *π*-electron-poor naphthalene diimide (NDI) into the polymer system, both modulus and fluorescence of the polymer films can be spatially regulated through the photodimerization of AN, which is controlled *in-plane* by photomasks, and becomes gradient in the *vertical* direction due to the filter effect of light. Therefore, the exposed sample displays a well-ordered complex pattern with the same topography as the applied photomask and 3D gradient change of fluorescence from red to green laterally across the layers simultaneously. The spatial cross-linking and CT interaction of the gradient layer can be controlled independently, which not only provides the reliability and reversibility of the topographical and fluorescence dual-pattern but also endows the possibility for tailoring the pattern with memory and self-healing. These characters of the dual-pattern with reversible topography and 3D fluorescence declare the clear applications in smart multiplex displays, memory, anticounterfeiting, visual detections, and so on.

## 1. Introduction

Construction of well-ordered microstructure with multiplex stereo or three-dimensional (3D) gradient information via spontaneous organization or direct writing (facile, single-step, noninvasive, and reversible approach) is fundamentally significant to multidisciplinary fields [1–11]. For example, surface patterns with topography and fluorescence can be used in the area of data or information display, storage, and transfer [12, 13]. The fluorescence patterns work as anticounterfeiting logos and intelligent displays [14], and the topographic patterns are fabricated on CD plates to record data or work as braille [15]. Hence, a system integrated with two characteristic parameters of the surface patterns, microarchitecture, and fluorescence are fantastic for a wide diversity of applications, such as multichannel display, visual bioassay, biosensors, 3D cell culture scaffolds, anticounterfeiting, and smart electronic devices. To achieve this goal, despite of many efforts on it, it is still difficult to obtain well-ordered micropatterns with 3D fluorescence and topography through one technological process. If the dual-pattern with topography and fluorescence can be fabricated synchronously and further tuned reversibly, the information capacity and security will be undoubtedly enhanced. Particularly, the reversible pattern with 3D fluorescence rather than the 2D fluorescence would substantially increase the information capacity for the spatial data storage in the vertical direction, which is very promising, but has not yet been reported. Therefore, to develop a simple and robust method of fabricating the surface patterns with reversible 3D fluorescence and topography is highly urgent and necessary but remains challenging.

As an alternative process for generating surface patterns, wrinkling and buckling of thin films can be harnessed to fabricate micro- and nanoscale structures with different geometries and dimensions [16–22]. Surface wrinkling occurs due to compressive force originating from the mechanical instability [16, 23–27]. Although surface wrinkling is a very convenient and cost-effective strategy to generate patterns, the surfaces always buckle or wrinkle into random patterns without preferential order because of the difficulty in controlling the surface instability. Taking spatial and temporal advantages of photo-cross-linking, we recently realized the controllable release of stress across the layers to produce the well-ordered patterns of wrinkle on the photosensitive film with the aid of photomasks [28]. This light direct-writing approach provides a competitive, noncontacting, and easy-operating strategy to fabricate surface patterns [29] and will open various interesting applications. Based on this approach, we regulate a gradient in the mechanical properties and fluorescence color simultaneously in the film, for the first time, to generate well-ordered surface dual-pattern with reversible 3D fluorescence and topography.

**Scheme 1:**
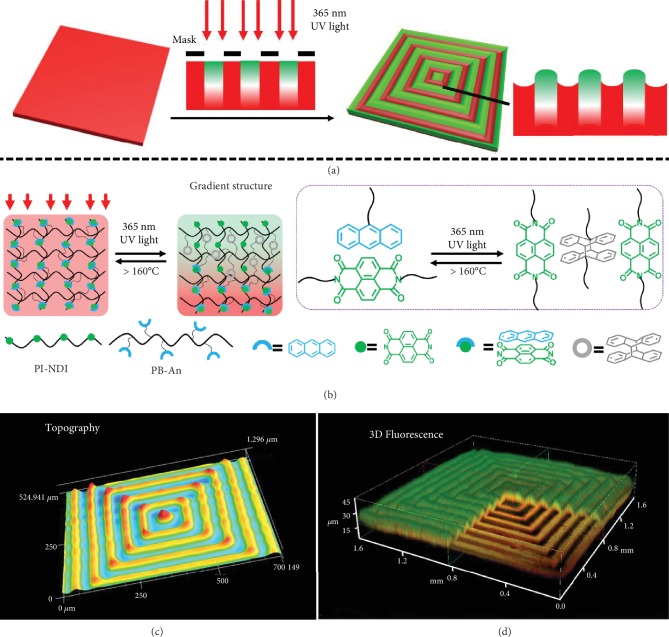
Schematic of patterns with 3D topography and 3D fluorescence, including (a) the direct-writing procedure of the dual-pattern on the film, (b) the involved chemical structures and dynamic reaction of the transformation between CT complex and dimers of anthracene, (c) the resulting 3D topography, and (d) the resulting 3D fluorescence images.

In detail, by introducing the charge transfer (CT) interaction [30, 31] between *π*-electron-rich AN [32, 33] and *π*-electron-poor NDI [34, 35] into the polymer film, we here demonstrate an example of surface dual-pattern with reversible topography and 3D fluorescence fabricated through the one-step light direct-writing approach, via tailoring a gradient in the mechanical properties and fluorescence color simultaneously in the photo-cross-linking film (Scheme 1). Both modulus and fluorescence of film are spatially regulated through photodimerization of AN [36–38], which are controlled *in-plane* by a photomask, and become gradient in the *vertical* direction due to the filtering effect of light. The gradient photodimerization induces a mechanical instability that can be used for direct-writing well-ordered patterns, simultaneously, and also leads to gradient fluorescence in the exposed regions changing from green to red in the *vertical* direction. Additionally, due to the reversibility of the photodimerization, the dual-pattern can be erased completely and other information can be rewritten. Interestingly, for the CT interaction, the resulting dual-pattern exhibits the pattern or shape memory and self-healing abilities at the microscale, which assures the durability of the patterns.

## 2. Results

### 2.1. Strategy for the Light Direct-Writing Dual-Pattern with Reversible Topography and 3D Fluorescence

Scheme 1 illustrates the whole strategy to produce well-ordered patterns with reversible topography and 3D fluorescence on the polymer film via the light direct-writing method. The key point in this strategy is to generate a gradient photo-cross-linking across the film, in which we can regulate the releasing of stress and the CT interaction across the gradient layer. Both the physical and chemical features are taken into consideration to design the photo-cross-linking system for the dual-pattern regulated by the light simultaneously. The AN-functionalized polymer PB-An (grafting ratio of AN is 10.2% of double bonds) is blended with the NDI-containing polyimide (PI-NDI) to fabricate film. The two main roles of NDI are to endow the obvious fluorescence and to form the physical cross-linking sites. Detailed information on the synthesis and characterization of these polymers can be found in Supporting Information (SI). Then, the polymer film covered by a photomask is exposed to 365 nm UV light to produce patterns. Before the UV light irradiation, the CT interaction between *π*-electron-rich AN and *π*-electron-poor NDI endues the red fluorescence to the film, while both AN and NDI units emit a blue-green fluorescence (Fig. [Supplementary-material supplementary-material-1]). Upon the irradiation, the dimerization of AN happens, leading to the cross-linking of the polymer chains and the destroying of the CT interaction between AN and NDI [39–41]. Due to the filter effect of surface photochemistry governed by the Lambert-Beer law, the gradient photodimerization of AN is generated in the *vertical* direction, giving rise to the gradient cross-linking and weakening of CT interaction from top layer to subsurface of the film. The red fluorescence emission of the CT complex turns green due to the photodimerization of AN. The photomask provides ability to *in-plane* control the photodimerization of AN. Thus, 3D fluorescence and modulus of the film can be spatially controlled through the one-step light irradiation in the presence of a photomask. After releasing compressive stress caused by the gradient cross-linking of film, the well-ordered wrinkles on the film with 3D fluorescence can be generated. Without the aid of photomasks, the disordered winkle is formed and induced by the gradient modulus and stress (Fig. [Supplementary-material supplementary-material-1]). When the gradient structure is formed regionally with the aid of masks, the compressive stress caused by gradient structure and modulus is regional and its releasing position can be controlled, which is responsible for the formation of the well-ordered complex patterns.

### 2.2. Controllable Topography of the Light Direct-Writing Dual-Pattern

**Figure 1:**
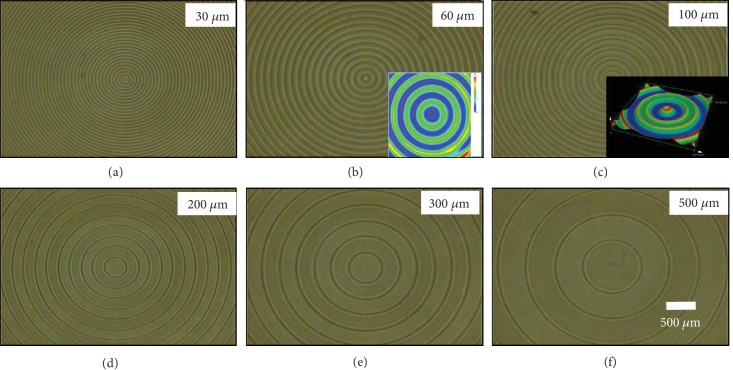
Optical microscope images of the patterns direct-written using a series of positive concentric circular annulus photomasks with different sizes: (a) 30 *μ*m; (b) 60 *μ*m; (c) 100 *μ*m; (d) 200 *μ*m; (e) 300 *μ*m; (f) 500 *μ*m. The thickness of polymer blend film is fixed at approximately 200 *μ*m. The intensity and exposure time of 365 nm UV light are approximately 50 mW/cm^2^ and 30 seconds, respectively.

**Figure 2:**
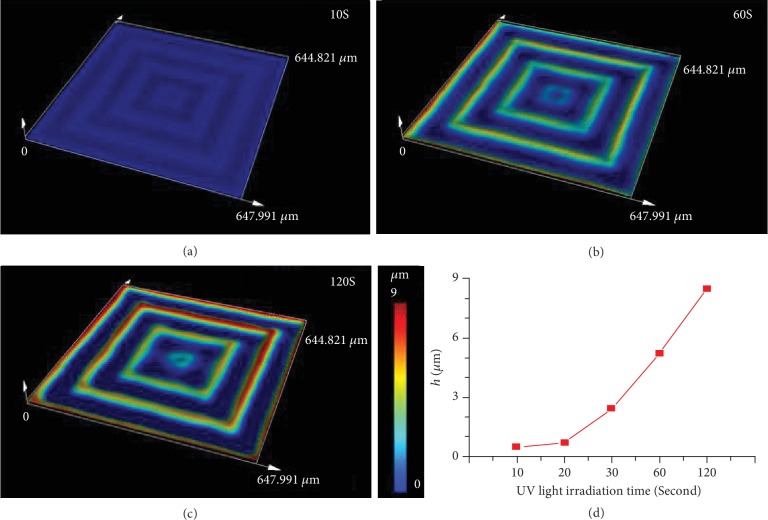
The evolution of the direct-written pattern from positive concentric square photomasks (size width/space: 50/50 *μ*m) with different exposure time observed with LSCM images: (a) 10 S; (b) 60 S; (c) 120 S. (d) The statistical height (*h*) of the patterns dependent on the exposure time. The thickness of the polymer blend film was ≈200 *μ*m. The intensity of 365 nm UV light is approximately 50 mW/cm^2^.

Due to the precise regulation of the mechanical properties and thus the release of stress across the gradient photo-cross-linked layer, a wide diversity of well-ordered complex patterns with controllable topography can be realized (Figures 1 and 2). To deeply understand the dynamic mechanism and gain well control, we investigated the pattern generation under various conditions. First, the effect of changing the pitch of the concentric circles on the photomask was explored. The masks with concentric circles in different sizes were designed and used to tune the resolution or wrinkle's wavelength (*λ*) of the patterns. As shown in Figure 1, a sequence of well-ordered patterns with the same morphologies as the photomasks can be easily written, and this new method allows us to prepare the surface patterns with the different periodicities. After irradiation under 365 nm UV light for 30 seconds, the patterns with *λ* varying from 30 to 500 *μ*m can be generated on the surfaces of films, indicating the well control and validity of this novel light direct-writing approach. In other words, the selective exposure via photomasks provides a top-down approach to precisely control the wavelength of wrinkles.

Then, the effect of 365 nm UV light exposure on the topography was investigated via controlling the irradiation time. We traced the growth dynamic of pattern under the irradiation of 365 nm UV light with a Laser Scan Confocal Microscope (LSCM). As shown in Figure 2, after irradiated by 365 nm UV light for only 10 seconds, the globally ordered pattern bearing the same morphology as the used photomask generates, and the height (*h*) of the resulting pattern is approximately 0.5 *μ*m. When the exposure time increased to 20 seconds, the *h* was increased to approximately 0.72 *μ*m. The *h* dramatically arrived to 8.52 *μ*m after irradiation for 120 seconds. Hence, the height of the patterns increases for the prolonging of the UV light exposure time, which can be ascribed to the controlled cross-linking density and the accompanying modulus fundamentally. The gradual process of the photodimerization reaction upon continuous irradiation of 365 nm UV light results in a noticeable but gradual change of cross-linking density dependent on the UV light exposure time (Fig. [Supplementary-material supplementary-material-1]), leading to the modulus mismatch and thus the generation of compress stress across the gradient layers. The controllable releasing of the stress induces the formation of well-ordered patterns. With the prolonging irradiation, the increased modulus and thickness of the stiff layer lead to the higher and more unbalanced compressive stress between unexposed and exposed regions, resulting in the faster growth of *h*. Meanwhile, the thermal effect of the chemical reaction is enhanced for the prolonging irradiation time, which is another factor for the growing of the patterns.

Furthermore, the high fidelity with sharp edges and corners is another important concern and technical requirement for complex 3D patterns. The masks with triangle and hexagon besides square in different sizes were also used in the light direct-writing process, and a series of topographies were achieved through this approach. It is shown (Fig. [Supplementary-material supplementary-material-1]) that the patterns can be written on the film surfaces and that the sharp edges and corners are all uniform and clear, which is hard to be achieved in the existing noncontact patterning technologies. Overall, in this convenient light direct-writing method, both wavelength and amplitude of the topography can be controlled by the UV light irradiation time and masks, and the patterns on the masks can be transferred to the film surface accurately. Hence, through this method, complicated and versatile patterns can be written on the films directly and conveniently without sophisticated technologies, potential for developing a general method in the area of multiplex information.

### 2.3. 3D Fluorescence of the Dual-Pattern

Due to the unique design of supramolecular chemistry feature integrated with light-controllable manipulation of gradient cross-linking in this polymer system, the 3D fluorescence pattern is formed simultaneously and can be controlled across the *vertical* direction, enabling the 3D gradient spectrographic information of the fluorescence pattern through a facile and noninvasive manner. As the other form of the dual-pattern, the fluorescence pattern increases the information capacity especially with the gradient fluorescence pattern in the *vertical* direction.

**Figure 3:**
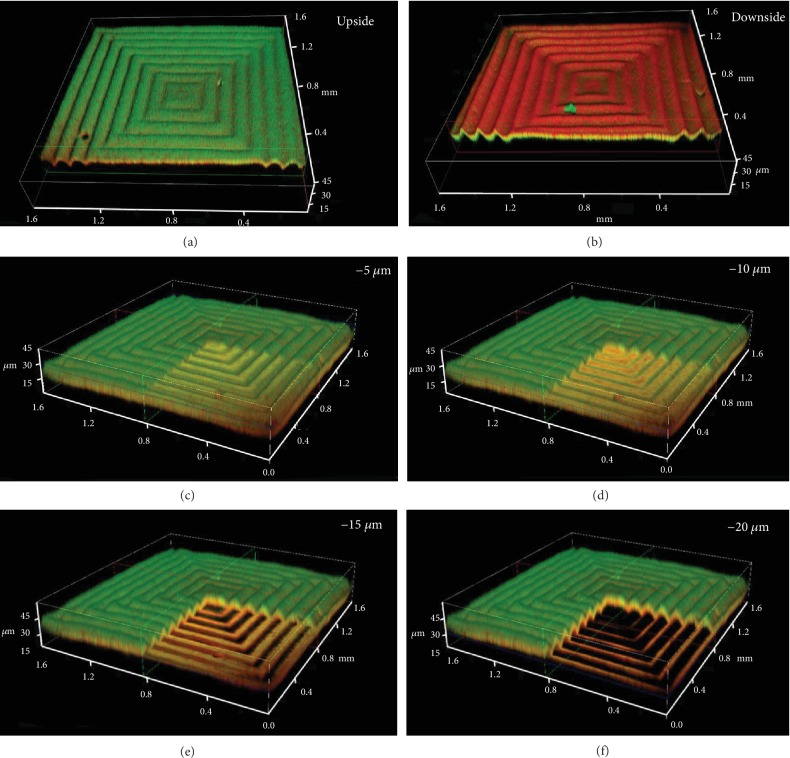
3D Fluorescence image of the dual-pattern obtained via the light direct-writing method. (a) Fluorescence image on the upside of the pattern; (b) fluorescence image on the downside of the pattern; (c-f) the fluorescence images and corresponding sectional fluorescence images of the dual-patterns at different depths of the film (5 *μ*m, 10 *μ*m, 15 *μ*m, and 20 *μ*m). The pattern was written with positive concentric square photomasks (size width/space: 50/50 *μ*m). The thickness of the polymer blend film was ≈200 *μ*m. The intensity and exposure time of 365 nm UV light were ≈50 mW/cm^2^ and 30 seconds, respectively.

After the exposure of 365 nm UV light, exposed regions of the as-prepared polymer film exhibited a unique green photoluminescence completely different from those of film at the unexposed region (Fig. [Supplementary-material supplementary-material-1]). Due to the photodimerization of AN, the CT complex between NDI and AN moieties with red fluorescence was destroyed, and the surface of the film turned into a cross-linked and rigid layer with green fluorescence from NDI. To gain detailed insight into the dependence of the fluorescence pattern upon the irradiation of 365 nm UV light, we traced the kinetics and fluorescence of the photodimerization of AN through UV-vis spectra (Fig. [Supplementary-material supplementary-material-1]) and fluorescence emission spectra (Fig. [Supplementary-material supplementary-material-1]), respectively. With the increasing 365 nm UV irradiation, the UV absorption peaks of AN decreased and the fluorescence emission at 650 nm assigned to the CT complex obviously was weakened simultaneously. It is no doubt that the photodimerization of AN formed the dimers and destroyed the CT complex between AN and NDI, thus weakening the red emission of the CT complex and releasing the noncomplexed NDI, which emits blue-green fluorescence [39, 42]. Visually, the micropatterns with 3D fluorescence was monitored by super-resolution multiphoton confocal microscopy (STED). As shown in Figure 3(a), the fluorescence of exposed regions on the top layer turns from red to green. On the other hand, the masked areas remained unchanged both in the modulus and in the CT interaction. Therefore, the sample at the unexposed areas exhibited the red fluorescence from the CT complex while at the exposed regions displayed a 3D gradient change of fluorescence from green to red laterally across the film. Therefore, the fluorescence patterns are formed not only on the surface but also in the 3D network for the filter effect of the UV light. The thickness of the gradient cross-linked layer was estimated with UV light transmittance (Fig. [Supplementary-material supplementary-material-1]), which was approximately 40 *μ*m where the UV light could reach. The cross-section of the pattern declares that fluorescence of the irradiated areas changes from green to white to the red gradually in the *vertical* direction ([Supplementary-material supplementary-material-1]), which also strongly evidenced that the well-ordered pattern is formed and originated from the gradient structure and stress. The fluorescence of the pattern turns to yellow-green at the depth of 5 *μ*m (Figure 3(c)). Going deeper, the orange-red fluorescence appears at 10 *μ*m (Figure 3(d)) and red fluorescence at 15 *μ*m (Figure 3(e)). The downside of the 3D pattern exhibits an inconspicuous pattern (Figure 3(b)), and the fluorescence of the exposed areas remains red for the absence of 365 nm UV light. Hence, it is convenient and possible to control the spatial gradient CT interaction to produce patterns with 3D fluorescence through this one-step approach. It should be noted that fluorescence pattern obtained in this system is very stable under ambient conditions due to the excellent stability of the AN dimers, which is rarely achieved for other fluorescence patterns with the photochromic molecules.

### 2.4. The Reversibility, Memory Behavior, and Healing Ability of the Dual-Pattern

**Figure 4:**
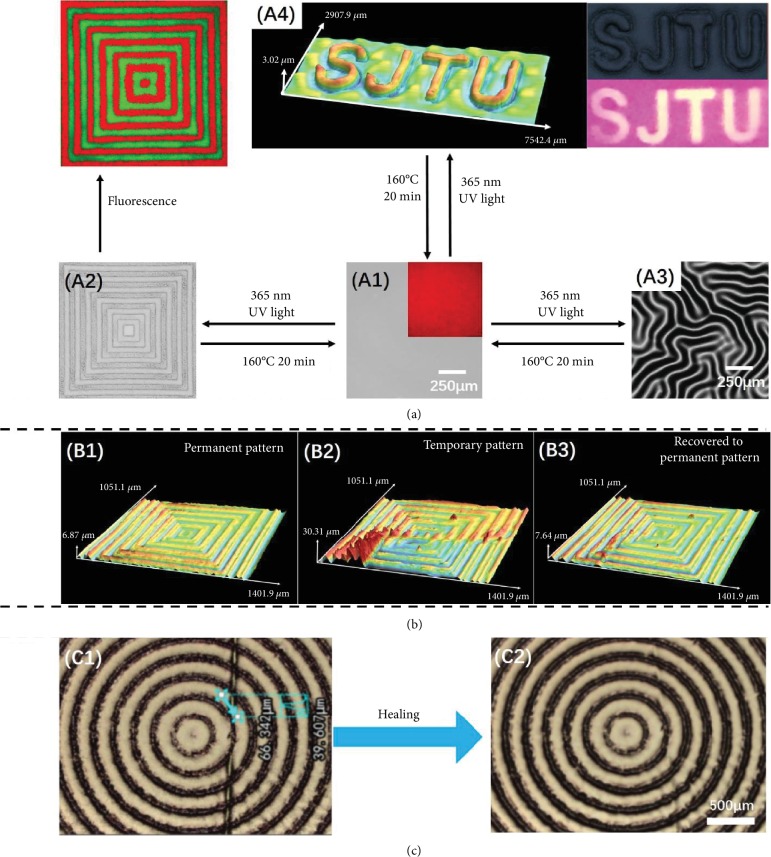
The reversibility, memory behavior, and healing ability of the dual-pattern. (a) The LSCM and fluorescence images showing the cyclic erasable process: writing the patterns, erasing the patterns, and rewriting the patterns: (a, I) topography and fluorescence image of the original sample; (a, II) topography and fluorescence image of the written sample with the patterns of concentric squares (size width/space: 50/50 *μ*m); (a, III) the topography of the written sample without the mask during the exposure; (a, IV) 3D topography and fluorescence of the written sample with the patterns of “SJTU.” The thickness of the polymer blend film was ≈200 *μ*m. The intensity and exposure time of 365 nm UV light were ≈50 mW/cm^2^ and 30 seconds, respectively. (b) LSCM images showing the shape memory process of the 3D well-ordered pattern: (b, I) the original shape of the pattern (concentric squares, size width/space: 50/50 *μ*m); (b, II) temporary state of the pattern; (b, III) the recovery shape of the pattern after heated at about 60°C for 3 minutes. (c) Optical images showing the self-healing process of the 3D pattern: (c, I) the damaged sample (concentric circular annulus (size width/space: 100/100 *μ*m)) with a crack; (c, II) the healed sample after heated at about 80°C for 5 minutes.

Since the key point to produce the wrinkle pattern is the photodimerization of AN, the well-ordered pattern can be tuned dynamically by controlling the reversible photodimerization, allowing us to write, erase, and rewrite various patterns. This is difficult to be achieved using the traditional patterning approaches such as photolithography and nanoimprint, in which the pattern is fixed at the final state and limited by the complicated technology itself. While, in this system, taking advantage of the chemical character of the polymer which is the reversibility of the AN dimerization, this kind of patterns written on the films via the UV light exposure could be erased via the thermal treatment at 160°C. As shown in Figure 4(a), the dual-pattern (Figure 4(a), II) was written on the polymer film through being irradiated with 365 nm UV light for 30 seconds covered with the mask. After the patterned sample (Figure 4(a), II) was heated at 160°C for 20 min, the pattern was erased for the dedimerization and the topography of the sample returned to the initial smooth state with red fluorescence. For the rewriting process, the erased sample was irradiated again covered by another mask with letters “SJTU” to rewrite pattern of SJTU (Figure 4(a), IV). The same procedures were taken on the sample with random wrinkle (Figure 4(a), III); the erasing and rewriting were also realized in the route of aI-aIII-aI-aII. Therefore, patterns with the topography and 3D fluorescence can be erased and the films can be reused in the information storage for the reversible dimerization of AN.

It is very interesting that this kind of dual-pattern exhibits memory behavior, and the topographic pattern with the temporary shape can recovery to the original shape through thermal treatment. As shown in Figure 4(b), firstly, the permanent pattern (with *h* about 6.9 *μ*m, Figure 4(b), I) was extruded and the temporary pattern was obtained with *h* about 30.3 *μ*m at the peak (Figure 4(b), II). Then, the sample with temporary pattern was heated at 60°C for 3 minutes. The pattern recovered to the permanent state (*h* about 7.6 *μ*m). In this process, it is the CT interaction that assures the pattern memory behavior. The CT interaction takes part in the formation of the film and works as the physical cross-linking site, which restricts the chain mobility especially in the uncross-linked layer. During the pattern recovering process, the energy from the thermal treatment ruins the physical cross-linking formed via the CT interaction and liberates the polymer chains and the patterns return to the stabilized permanent state, while the thermal treatment temperature for pattern recovery is not high enough to break the covalent bonds (dimers of AN) and the patterns cannot be ruined. Hence, the dual-pattern exhibits pattern memory behavior in the microsize without ruining the pattern, which is virtually impossible and has not been presented in the existing literatures as we know. With the pattern shape memory ability, even if the patterns' topography is crumpled for the compressive force, the patterns can recovery to original state and the information and data will be retained.

The resulting dual-pattern also exhibits the performance of self-healing ability due to the presence of the CT interaction between AN and NDI in the film. As shown in Figure 4(c), a crack ~30-40 *μ*m was created on the patterned surface by a knife. After heated at 80°C for 5 min, the crack was healed, while the control sample (PB-An) without the CT interaction cannot be healed even after 10 minutes of heating at 80°C (Fig. [Supplementary-material supplementary-material-1]). It should be noted that this temperature is below the dedimerization temperature of AN's dimers and the microcosmic patterns or structures could be healed without ruining the patterns. Most of the cases, the surface pattern will be erased because of the chain movement which happens during the healing process [28]. However, in this system, the crack is healed without ruining the pattern, which might be ascribed to the different chemical mechanisms and interactions for the formation of the patterns and the healing behavior. The patterns are written on the film via the dimerization of AN, and it calls for the temperature higher than 160°C to trigger the dedimerization and ruin the patterns, while it is the dynamical noncovalent CT interaction that realizes the self-healing behavior. In the healing process, due to the noncovalent CT interaction, some chains can be liberated and relinked when the temperature is high enough (about 80°C) but much lower than the AN dedimerization temperature, and the pattern is healed. Hence, for the crack that is healed via the CT interaction rather than the AN dimerization (the chemical mechanism for the patterns' formation), the patterns are not necessary to be ruined in the healing process.

**Figure 5:**
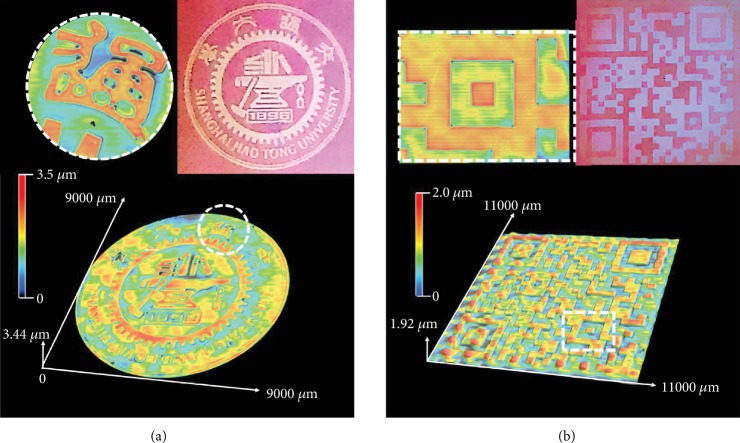
Laser scanning confocal microscope (LSCM) images showing the complex patterns obtained via the light direct-writing method. (a) 3D school badge of Shanghai Jiao Tong University. The intensity and exposure time of 365 nm UV light were ≈50 mW/cm^2^ and 30 seconds, respectively. (b) 3D “QR Code” with fluorescence under UV light. The thickness of the polymer blend film was ≈200 *μ*m. The intensity and exposure time of 365 nm UV light were ≈50 mW/cm^2^ and 25 seconds, respectively.

The pattern memory behavior and healing ability of the dual-pattern declare the stability and durability of the patterns. On the other hand, the patterns can be erased thoroughly and rewritten with other information at a higher temperature (>160°C, dedimerization temperature of the AN dimers), which displays recycle ability. No doubt that our one-step manufacturing dual-pattern with these characters can serve well for the development of a new type of smart materials and surfaces that have clear applications in smart displays, message storage, anticounterfeiting, and visual bioassays. Also, this kind of noncontact direct-writing technology affords convenience and possibilities in the construction of well-ordered and controlled dual-pattern with multiplex and complex information. As shown in Figure 5, the complex patterns with the diverse topographies can be written on the films clearly with 3D fluorescence. In Figure 5(a), the school badge of Shanghai Jiao Tong University was direct-written on the film surface; the 3D “school badge” displayed topography with the height about 3.4 *μ*m. The locally enlarged views are provided to show the pattern's details. The fluorescence pattern of “school badge” came out under the UV light. Similarly, a more complicated pattern of the 3D “QR Code” with fluorescence pattern could be also obtained through the light direct-writing method (Figure 5(b)). Hence, it is declared that various and complicated 3D patterns can be achieved in this method. This facile approach exhibited the potential for light directing any desired complex pattern with high fidelity of the applied mask, promising for a general method for micropattern. Moreover, this kind of patterns possessing more high-throughput information of reversible topography and 3D fluorescence occupy the absolute advantage over the 2D patterns in the area of identification and information transferring for the appreciable feel in touch.

## 3. Discussion

We demonstrated a novel, convenient, one-step, and reversible method of light-induced direct writing for the complex well-ordered patterns with topography and 3D fluorescence. Based on these unique advantages of the general and robust approach, and integrated design with features of the CT interaction and the reversible photodimerization, we firstly achieved the 3D complex architecture with well-ordered topography and 3D gradient fluorescence pattern, which is very difficult to be fabricated using traditional methods. Various complicated and well-ordered patterns with controllable morphology can be obtained through this method conveniently, and the size and resolution of the patterns can be precisely tuned by the UV exposure time and pitch of the applied mask, potential for a general method for micropattern. Moreover, due to the nature of dynamic chemistry, the ordered pattern also can be erased and rewritten. Meanwhile, the CT interaction in the network assures the memory behavior and self-healing ability of the patterns at the microscale, which provides the durability of the patterns. This general, operable, and noncontact approach exhibits clear applications in smart multiplex displays, memory, anticounterfeiting, and visual detections.

## 4. Materials and Methods

### 4.1. Materials

N,N-Dimethylformamide, 9-anthracenemethanol, toluene, poly(ether diamine) (Jeffamine D230), and 4-methylbenzenesulfonic acid were purchased from Adams-Beta Co. Ltd. (Shanghai, China). 2-Mercaptoethanol and succinic anhydride were purchased from J&K Scientific Ltd. Chloroform, polybutadiene (PB) (Mw ∼ 200000), and 1,4,5,8-naphthalenetetracarboxylic dianhydride were purchased from Sigma-Aldrich. All the reagents were used as received.

### 4.2. Fabrication of the Dynamic Well-Ordered Dual-Pattern through the Light Direct-Writing Method

The PB-An and PI-NDI (at the weight ratio of 5 : 1) were dissolved in toluene, and the solution was cast on the glass plate. Then, the films were heated at approximately 80°C for 12 h to evaporate solvent. To write the patterns, the films were exposed to 365 nm UV light covered with a photomask for a specific time to initiate the photodimerization of AN obtaining a well-ordered surface pattern that transferred the pattern on the mask. To erase the pattern, the film was heated at 160°C. The regeneration of patterned films could be realized in the procedure.

### 4.3. Characterization

^1^H NMR spectra were recorded on a Varian Mercury Plus spectrometer (500 MHz) with chloroform-d (CDCl_3_) as the solvent and tetramethylsilane (TMS) as an internal standard at room temperature. Optical microscope images of the patterns were obtained with profile measurement microscope (VF-7501, KEYNCE, Japan). The UV-vis spectra were carried out with TU-1091 spectrophotometry (PerkinElmer, China). The fluorescence spectra were recorded on an LS-55B fluorescence meter (PerkinElmer, Inc., USA), and the excitation wavelength was 365 nm. The 3D fluorescence images of the patterns were observed with a super-resolution multiphoton confocal microscope. The observation of the pattern topography was performed on the laser scanning confocal microscopy LSCM (LEXT OLS4100, Olympus, Japan) and laser profilometer (VF-7510, Keyence, Japan).

## Data Availability

All relevant data are included in the paper and supplementary information files.

## Supplementary Materials

Supplementary Materials Synthesis of the hydroxyl-functional polybutadiene via the thiol-ene click reaction. Synthesis of the anthracene-grafted polybutadiene (PB-An). Synthesis of NDI Contained PI (PI-NDI). Fig. S1: synthesis process of hydroxyl-functional polybutadiene (PB-OH) and the anthracene-grafted polybutadiene (PB-An). Fig. S2: chemical structure and 1H NMR spectrum of PB-OH. Fig. S3: chemical structure and 1H NMR spectrum of PB-An. Fig. S4: chemical structure and 1H NMR spectrum of PI-NDI. Fig. S5: fluorescence spectra of (A) PB-An (B) PI-NDI. Fig. S6: UV-vis transmittance spectra of polymer blend (PB-An/NDI-230 = 4 : 1) dependent on the film thickness. Fig. S7: LSCM image showing the disordered wrinkle in the absence of photomask. Fig. S8: UV-vis spectral traces for the process of photodimerization of anthracene groups in the polymer blend film under irradiation by 365 nm UV light for different times. Fig. S9: LSCM images showing patterns written with different geometric configuration controlled with masks. Fig. S10: (A) trace of fluorescence emission spectra of a PB-An/PI-NDI film for different irradiation times of 365 nm UV light. (B) Photograph of the film which was regional irradiated by 365 UV light for 10 minutes taken under 365 nm UV light. Fig. S11: laser scanning confocal microscope (LSCM) images of the control sample (without CT interaction) after heated for 10 minutes at 80°C. Movie S1: 3D fluorescence of the dual-pattern.
