# Supplementary material for: Light-Written Reversible 3D Fluorescence and Topography Dual-Pattern with Memory and Self-Healing Abilities
Source: Research (Wash D C). 2019 Nov 2;2019:2389254. doi: 10.34133/2019/2389254 (PMC6946259; doi:10.34133/2019/2389254)
Supplement: Supplementary Materials — Synthesis of the hydroxyl-functional polybutadiene via the thiol-ene click reaction. Synthesis of the anthracene-grafted polybutadiene (PB-An). Synthesis of NDI Contained PI (PI-NDI). Fig. S1: synthesis process of hydroxyl-functional polybutadiene (PB-OH) and the anthracene-grafted polybutadiene (PB-An). Fig. S2: chemical structure and 1H NMR spectrum of PB-OH. Fig. S3: chemical structure and 1H NMR spectrum of PB-An. Fig. S4: chemical structure and 1H NMR spectrum of PI-NDI. Fig. S5: fluorescence spectra of (A) PB-An (B) PI-NDI. Fig. S6: UV-vis transmittance spectra of polymer blend (PB-An/NDI-230 = 4 : 1) dependent on the film thickness. Fig. S7: LSCM image showing the disordered wrinkle in the absence of photomask. Fig. S8: UV-vis spectral traces for the process of photodimerization of anthracene groups in the polymer blend film under irradiation by 365 nm UV light for different times. Fig. S9: LSCM images showing patterns written with different geometric configuration controlled with masks. Fig. S10: (A) trace of fluorescence emission spectra of a PB-An/PI-NDI film for different irradiation times of 365 nm UV light. (B) Photograph of the film which was regional irradiated by 365 UV light for 10 minutes taken under 365 nm UV light. Fig. S11: laser scanning confocal microscope (LSCM) images of the control sample (without CT interaction) after heated for 10 minutes at 80°C. Movie S1: 3D fluorescence of the dual-pattern. [file 2389254.f1.zip › SI.docx]

Supplementary Materials

Light-Written Reversible 3D Fluorescence and Topography Dual-Pattern with Memory and Self-Healing Abilities

Jing Bai, Luzhi Zhang, Honghao Hou, Zixing Shi, Jie Yin, Xuesong Jiang*

**This file includes:**

Synthesis of the hydroxyl-functional polybutadiene via the thiol-ene click reaction

Synthesis of the anthracene-grafted polybutadiene (PB-An)

Synthesis of NDI Contained PI (PI-NDI)

Fig. S1 Synthesis process of hydroxyl-functional polybutadiene (PB-OH) and the anthracene-grafted polybutadiene (PB-An).

Fig. S2 Chemical structure and ^1^H NMR spectrum of PB-OH

Fig. S3 Chemical structure and ^1^H NMR spectrum of PB-An

Fig. S4 Chemical structure and ^1^H NMR spectrum of PI-NDI

Fig. S5 Fluorescence spectra of (A) PB-An (B) PI-NDI

Fig. S6 UV-vis transmittance spectra of polymer blend (PB-An/NDI-230=4:1) dependent on the film thickness

Fig. S7 LSCM image showing the disordered wrinkle in the absence of photomask.

Fig. S8 UV-vis spectral traces for the process of photo-dimerization of anthracene groups in the polymer blend film under irradiation by 365 nm UV light for different time

Fig.S9 LSCM images showing patterns written with different geometric configuration controlled with masks

Fig. S10 (A) Trace of fluorescence emission spectra of a PB-An/PI-NDI film for different irradiation times of 365 nm UV light. (B) Photograph of the film which was regional irradiated by 365 UV light for 10 minutes taken under 365 nm UV light.

Fig. S11 Laser scanning confocal microscope (LSCM) images of the control sample (without CT interaction) after heated for 10 minutes at 80℃

Movie S1 3D fluorescence of the dual-pattern

**Synthesis of the hydroxyl-functional polybutadiene via the thiol-ene click reaction**

To provide the grafting site for the anthracene group, the hydroxyl group was firstly grafted to the polybutadiene chains via the thiol-ene reaction (Fig. S1). Polybutadiene and 2-Mercaptoethanol were dissolved in toluene with trace amounts of the photoinitiator I907. The addition of 2-Mercaptoethanol was fixed at 20 % of the double bonds on polybutadiene chains. The solution was irradiated with 365 nm UV light for 12 h at room temperature and the obtained hydroxyl-functional polybutadiene was named as PB-OH. The reacted degree of hydroxyl groups is about 98 % for the addition of 2-Mercaptoethanol from the ^1^H NMR as shown in Fig. S2, and the grafting process is high efficiency for the click reaction.

**Synthesis of the anthracene-grafted polybutadiene (PB-An)**

The anthracene-grafted polybutadiene (PB-An) were synthesized according to Scheme S1. PB-OH and An-COOH were dissolved in toluene and the solution was stirred and refluxed for 12 hours. An-COOH was prepared according to our previously work (*1*). The addition of An-COOH was designed according to the hydroxyl group content of PB-OH. In this system, addition of An-COOH was 60 % of the hydroxyl groups grafted onto the PB-OH chains. The amount of anthracene moieties was quantified by both UV-vis and ^1^H NMR spectra. In this work, the grafting ratio of anthracene was about 52% of the hydroxyl groups.

**Synthesis of NDI Contained PI (PI-NDI)**

1,4,5,8-naphthalenetetracarboxylic dianhydride (2.68 g, 10 mmol) and Jeffamine D-230 (2.3 g, 10 mmol) were dissolved in DMAc (40 mL), and the mixture was heated at 135 °C for 24h. Then the dark solution was into diethyl ether to get the solid-state crude product. After washing for three times, the product was vacuum-dried at 80 °C overnight.





Fig. S1 Synthesis process of hydroxyl-functional polybutadiene (PB-OH) and the anthracene-grafted polybutadiene (PB-An).


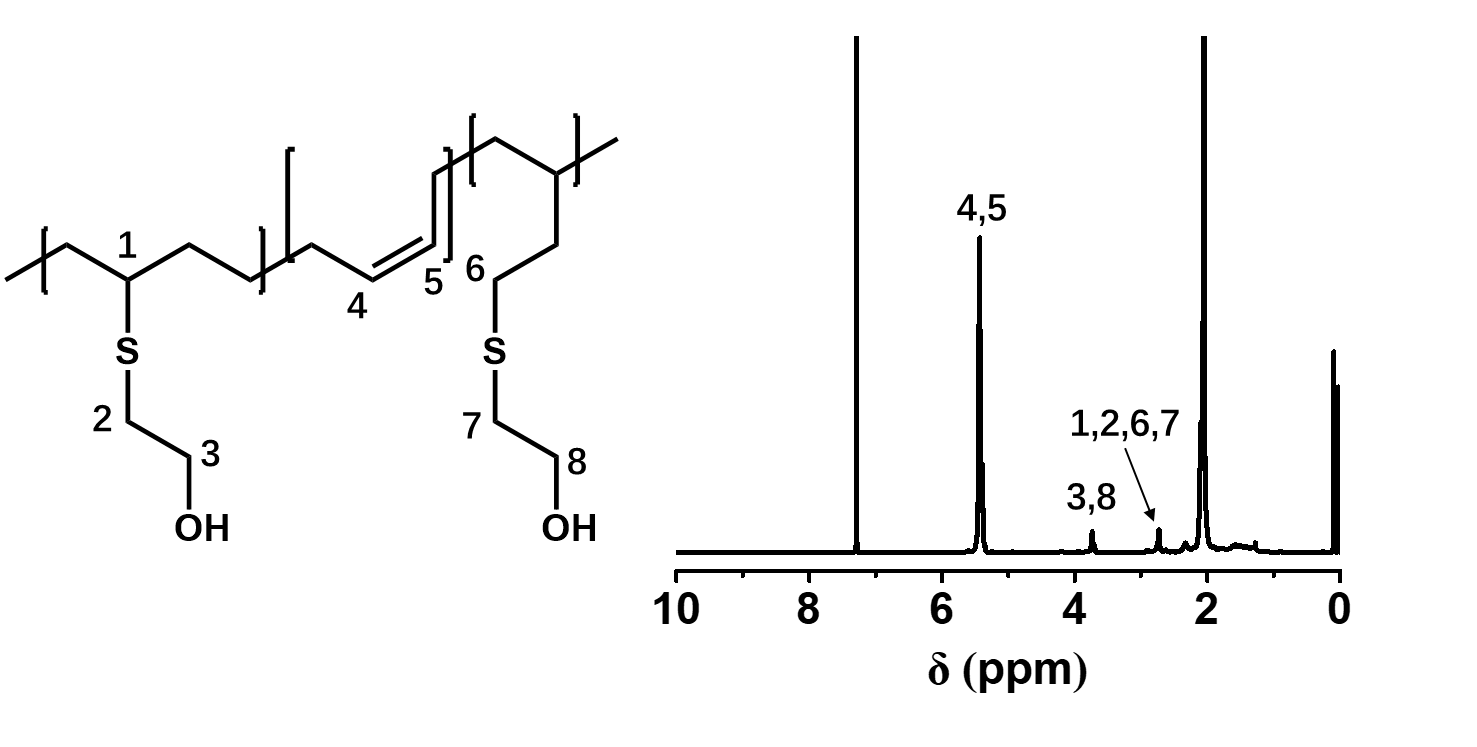


Fig. S2 Chemical structure and ^1^H NMR spectrum of PB-OH


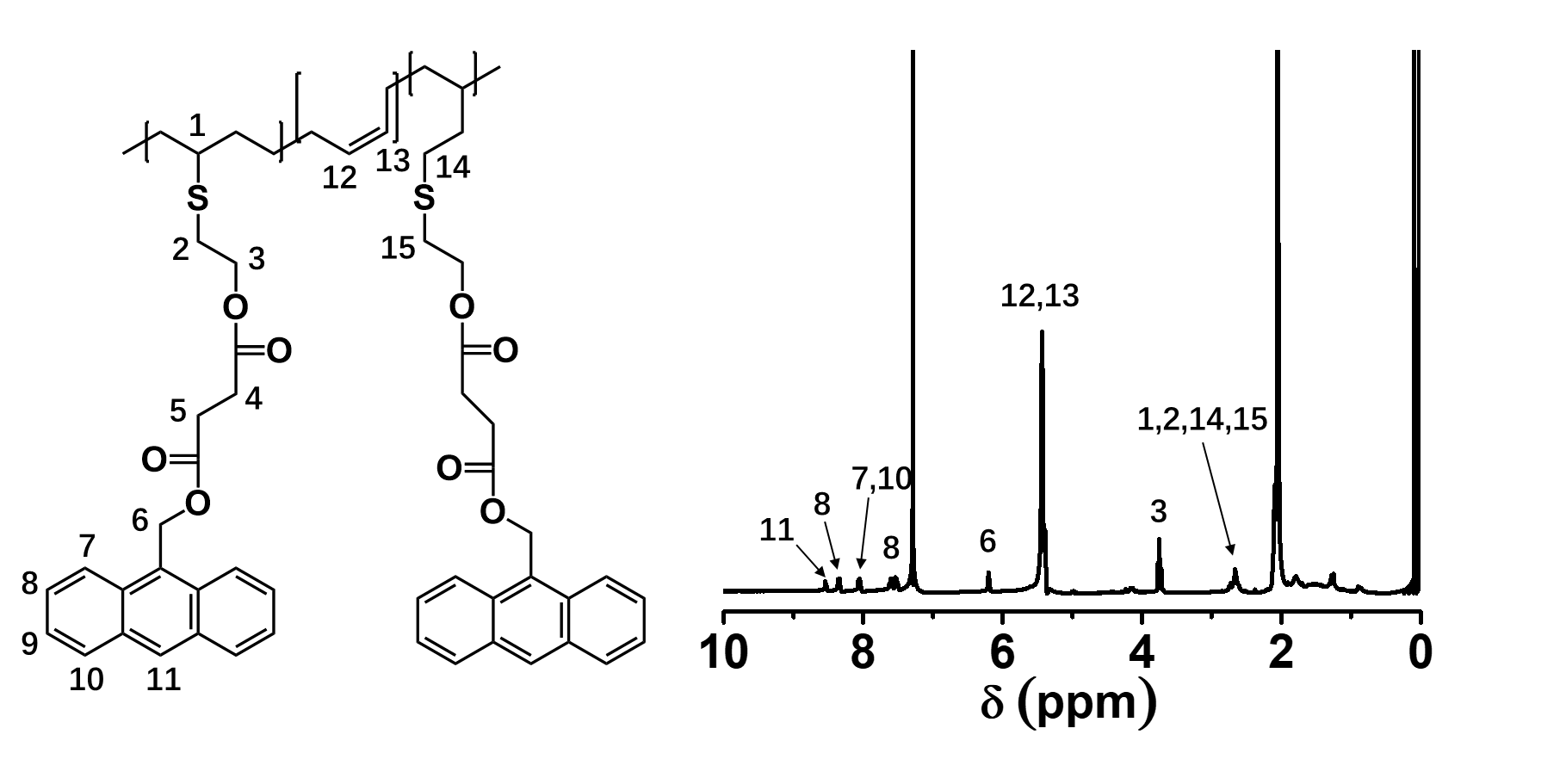


Fig. S3 Chemical structure and ^1^H NMR spectrum of PB-An


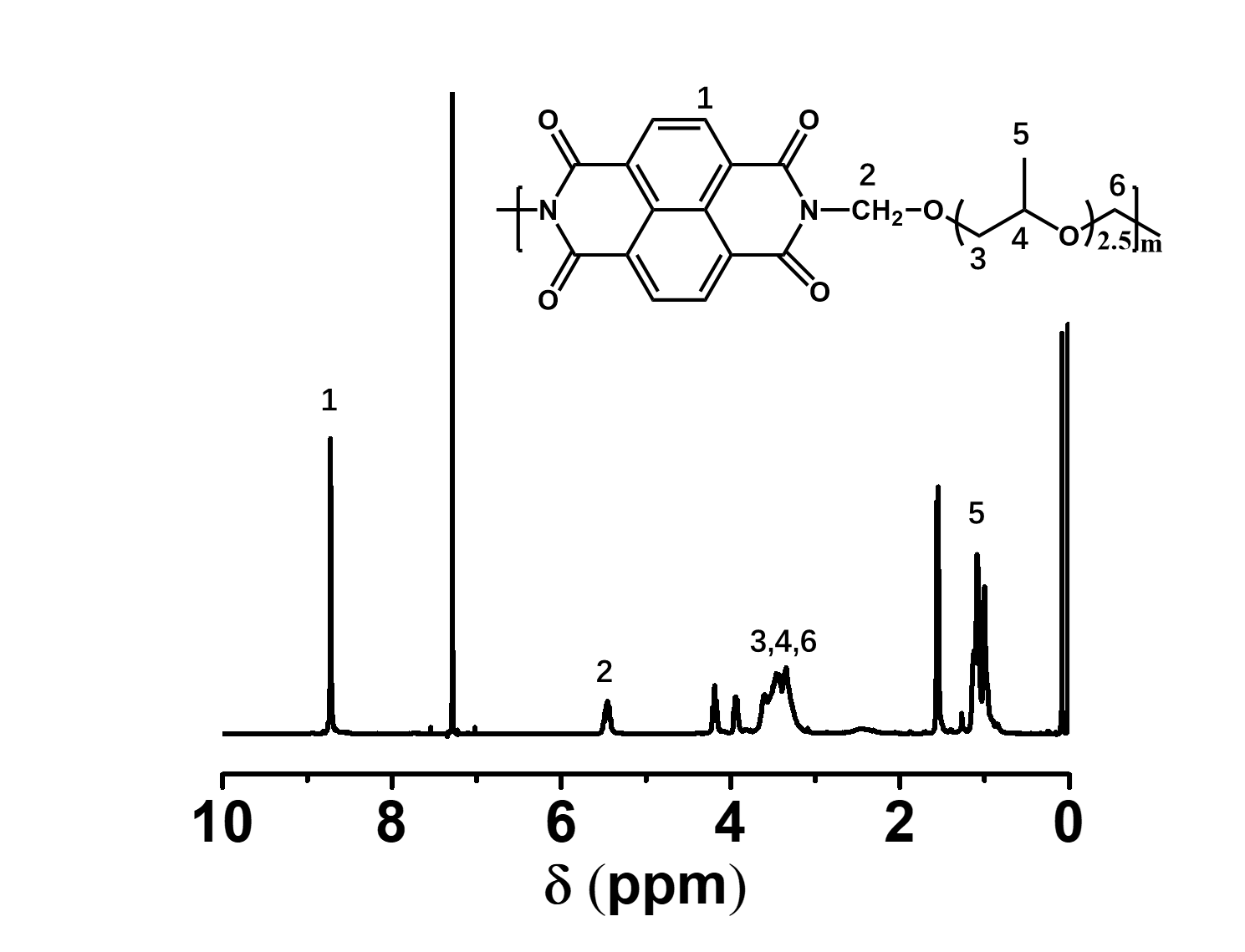


Fig. S4 Chemical structure and ^1^H NMR spectrum of PI-NDI


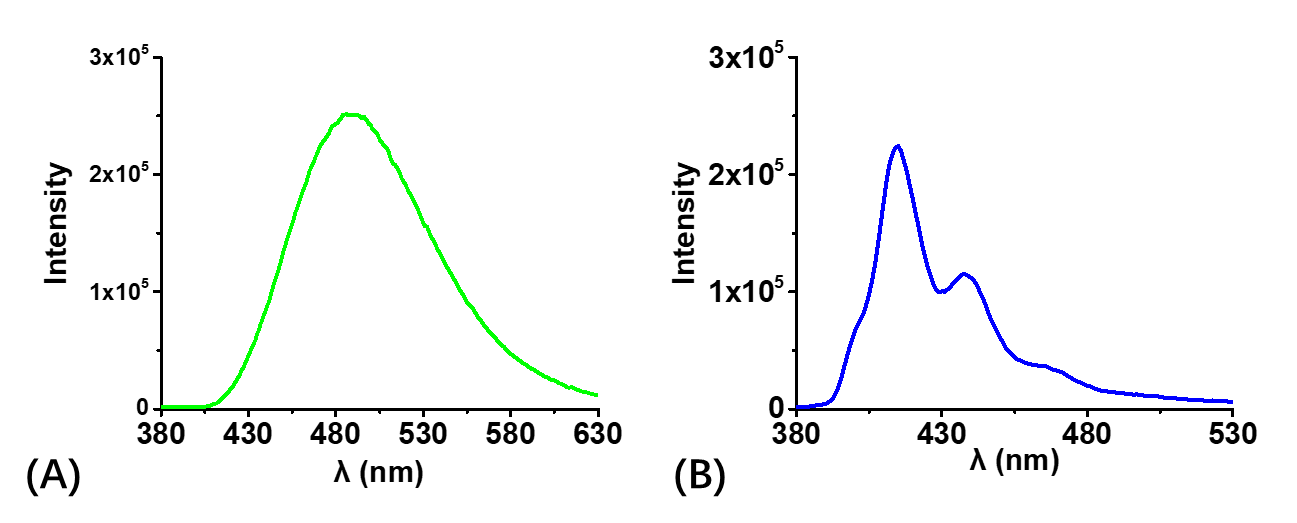


Fig. S5 Fluorescence spectra of (A) PB-An and (B) PI-NDI

**The calculation of the gradient layer thickness**

In this system, the pattern was formed via the wrinkle mechanism driven by the gradient structure or stress. Therefore, it is important to calculate the thickness of the gradient layer. Anthracene processes typical absorption peaks whose intensities change with the UV light irradiation, and the un-irradiated part of the film has low transmittance (around 300-400) nm due to the absorption of anthracene, while the cross-linked layer shows high transmittance for the dimers have almost no absorption. Therefore, the UV light transmittance of the films with different thickness was traced to estimate the thickness of the gradient-crosslinked layer. As shown in Fig. S6, the transmittance of UV light decreased with the thickness, and the induced dimerization ratio of anthracene attenuated. Hence, the gradient structure was formed.





Fig. S6 UV-vis transmittance spectra of polymer blend (PB-An/NDI-230=4:1) dependent on the film thickness


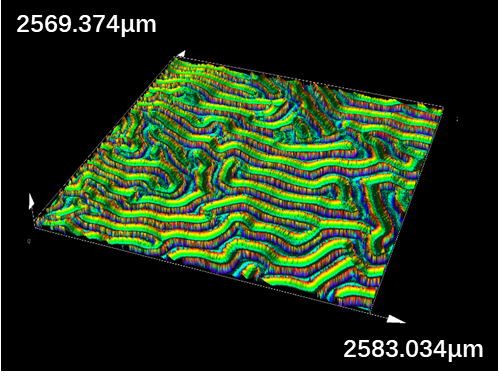


Fig. S7 LSCM image showing the disordered wrinkle in the absence of photomask.





Fig. S8 UV-vis spectral traces for the process of photo-dimerization of anthracene groups in the polymer blend film under irradiation by 365 nm UV light for different time


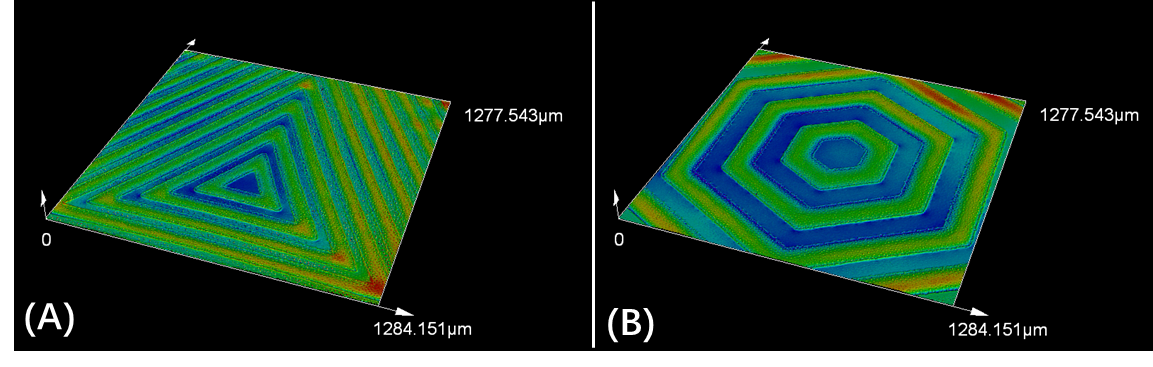


Fig.S9 LSCM images showing patterns written with different geometric configuration controlled with masks.(A) positive and negative hybrid concentric triangles (size width/space: 50/50 μm); (B) positive and negative hybrid concentric hexagons (size width/space: 100/100 μm). The thickness of the polymer blend film was ≈200 μm. The intensity and exposure time of 365 nm UV light were ≈50 mW/cm^2^ and 30 seconds, respectively.


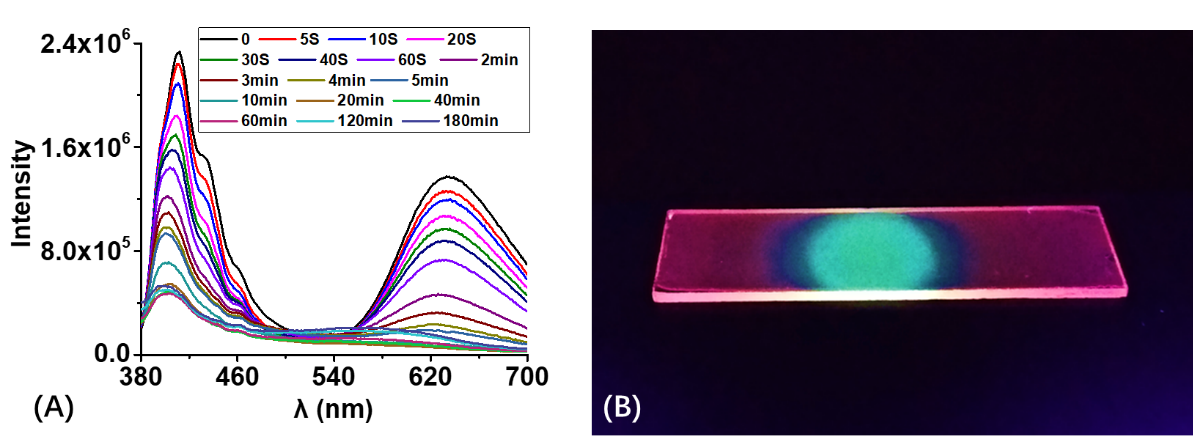


Fig. S10 (A) Trace of fluorescence emission spectra of a PB-An/PI-NDI film for different irradiation times of 365 nm UV light. (B) Photograph of the film which was regional irradiated by 365 UV light for 10 minutes taken under 365 nm UV light.


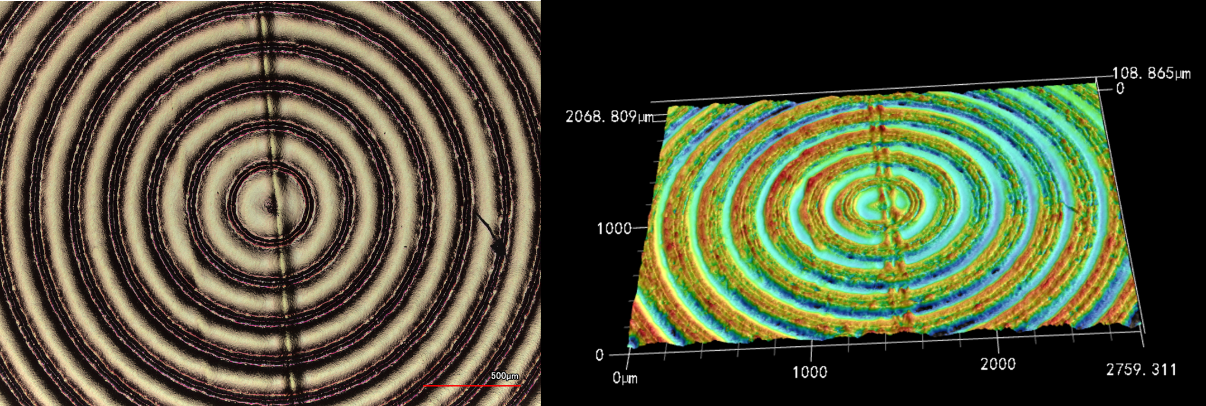


Fig. S11 LSCM images of the control sample (without CT interaction) after heated for 10 minutes at 80 °C.

**Reference**

1. J. Bai, Z. Shi, Shape Memory: An Efficient Method to Develop the Latent Photopatterned Morphology for Elastomer in Two/Three Dimension. *ACS Macro Letters* **6**, 1025-1030 (2017).
